# Supplementary material for: Transglutaminase 2-Mediated p53 Depletion Promotes Angiogenesis by Increasing HIF-1α-p300 Binding in Renal Cell Carcinoma
Source: Int J Mol Sci. 2020 Jul 17;21(14):5042. doi: 10.3390/ijms21145042 (PMC7404067; doi:10.3390/ijms21145042)
Supplement: Supplementary file 1 [file ijms-21-05042-s001.pdf]

Supplementary Figure 1

A

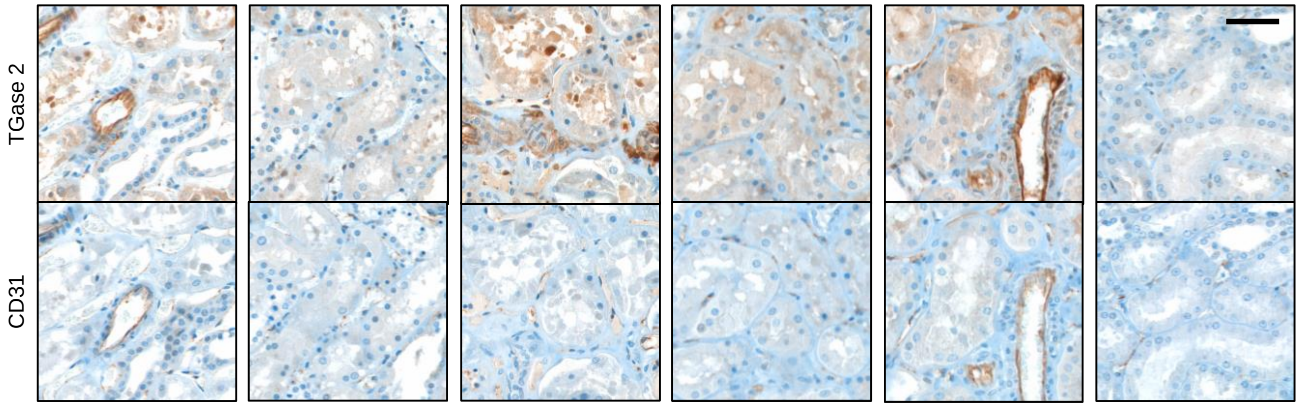

Scale bar = 50 μm.

B

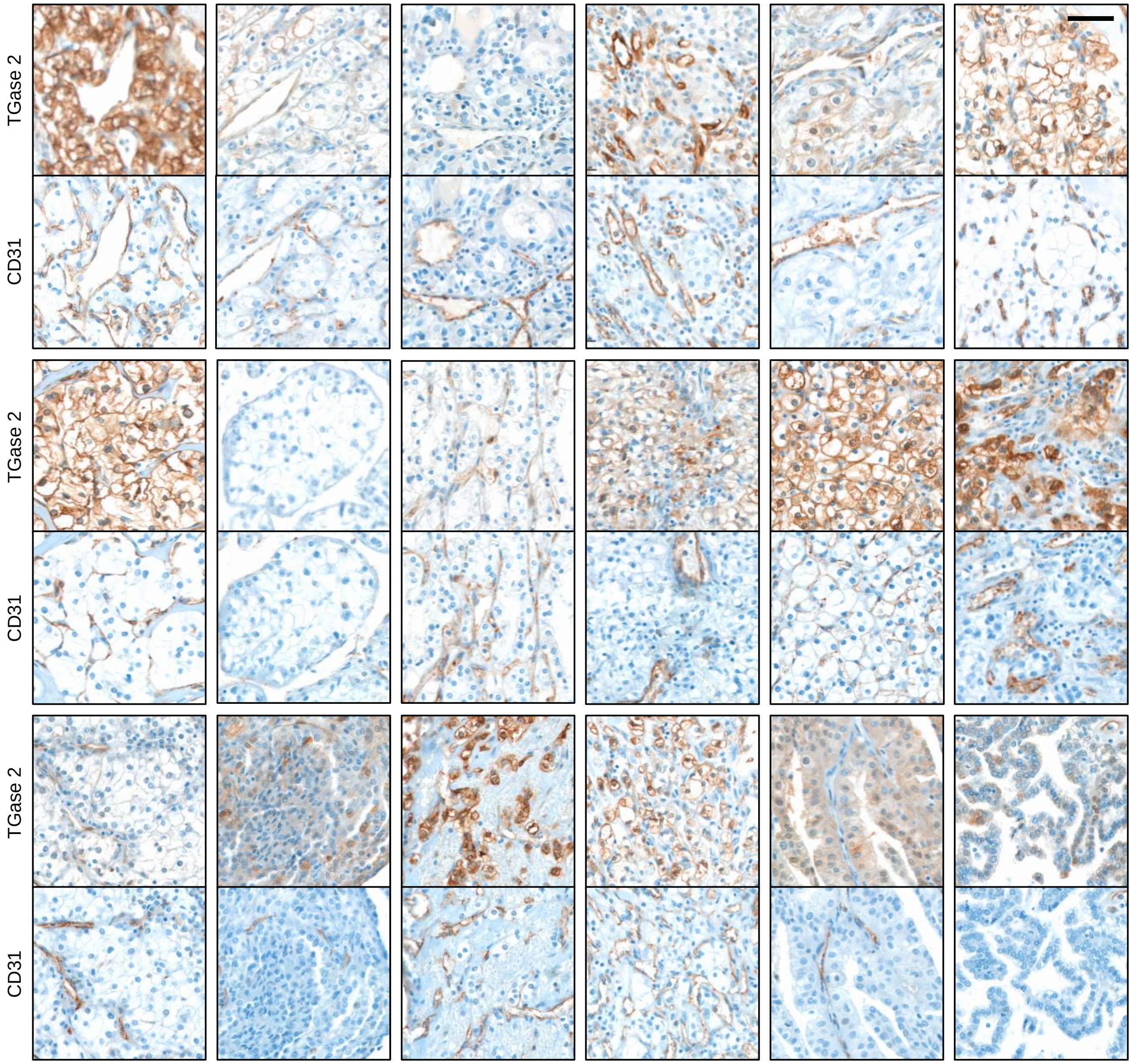

Scale bar = 50 μm.

Supplementary Figure 1

B (continued)

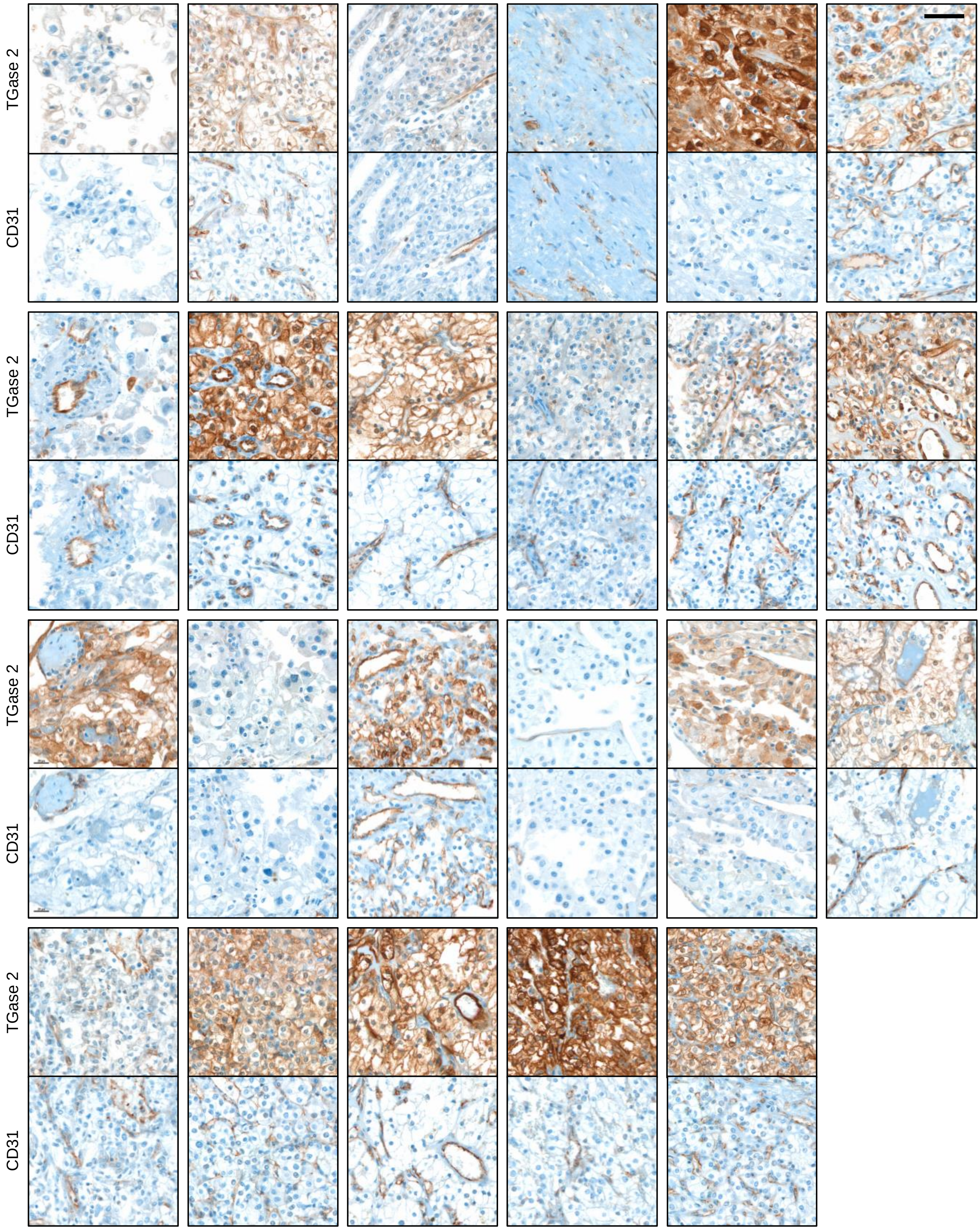

Supplementary Figure 1. Immunohistochemical staining of TGase 2 and CD31 in a human clear cell renal cell carcinoma tissue microarray.
(A) Image of TGase 2 (Top) and CD31 (Bottom) staining in human normal kidney tissues. (B) Image of TGase 2 (Top) and CD31 (Bottom) staining in human clear cell renal cell carcinoma tissues. Scale bar = 50 µm.

Supplementary Figure 2

Figure 2A

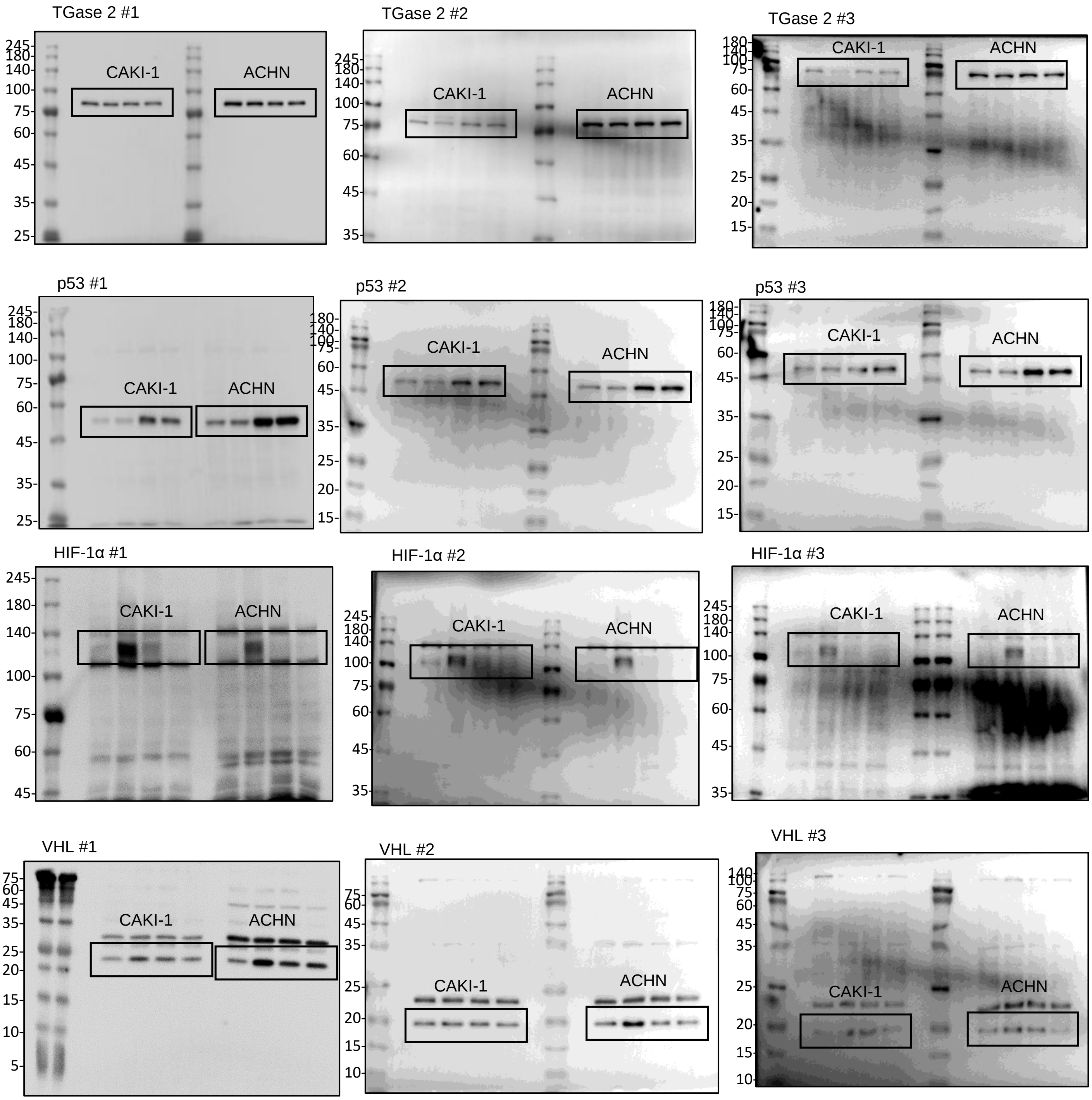

Supplementary Figure 2. Full gel images of western blotting

Supplementary Figure 2

Figure 2A

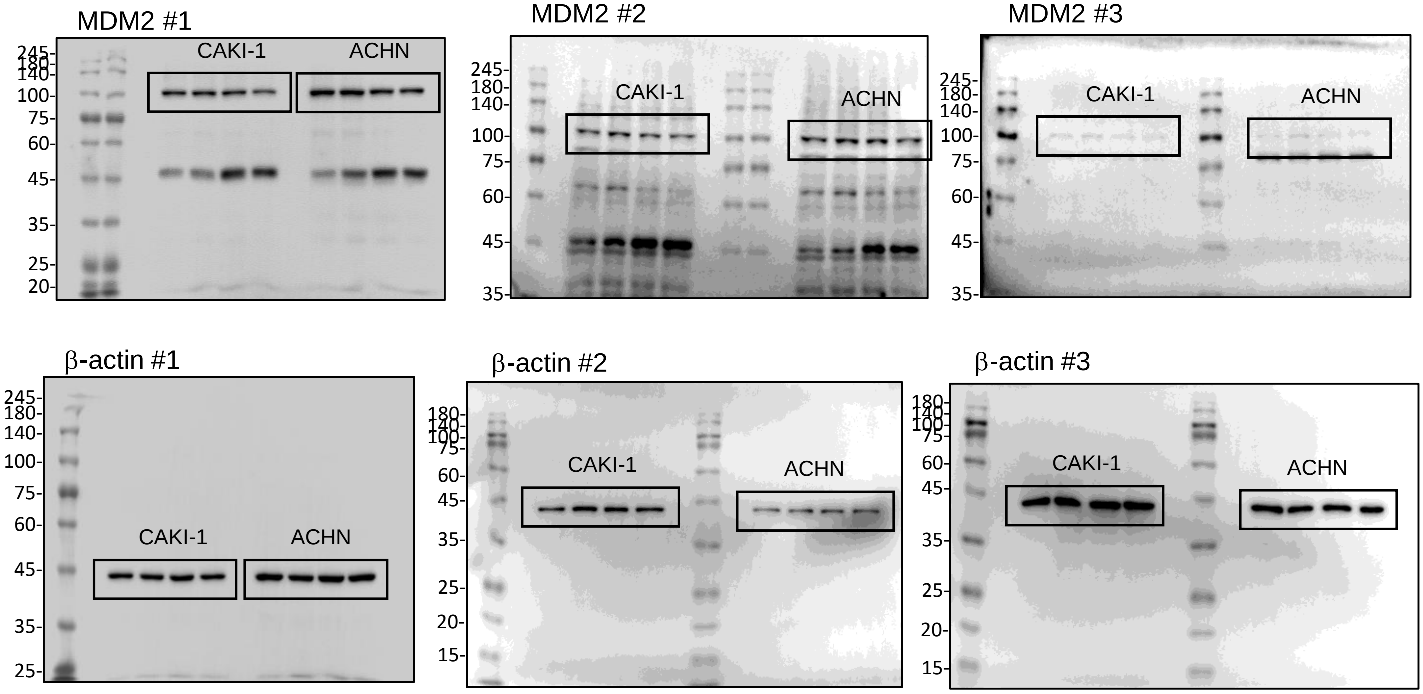

Supplementary Figure 2. Full gel images of western blotting

Supplementary Figure 2

Figure 2C

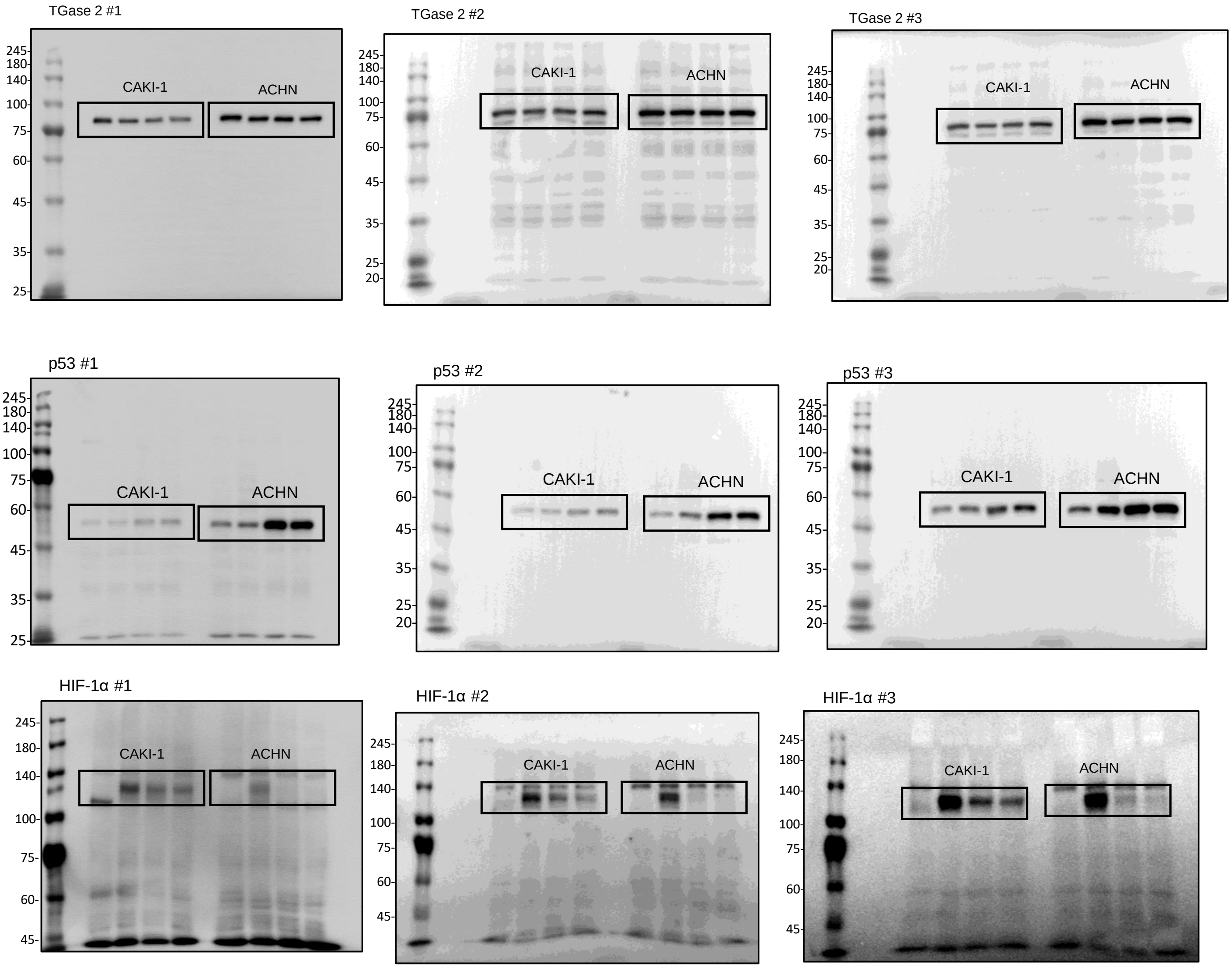

Supplementary Figure 2. Full gel images of western blotting

Supplementary Figure 2

Figure 2C

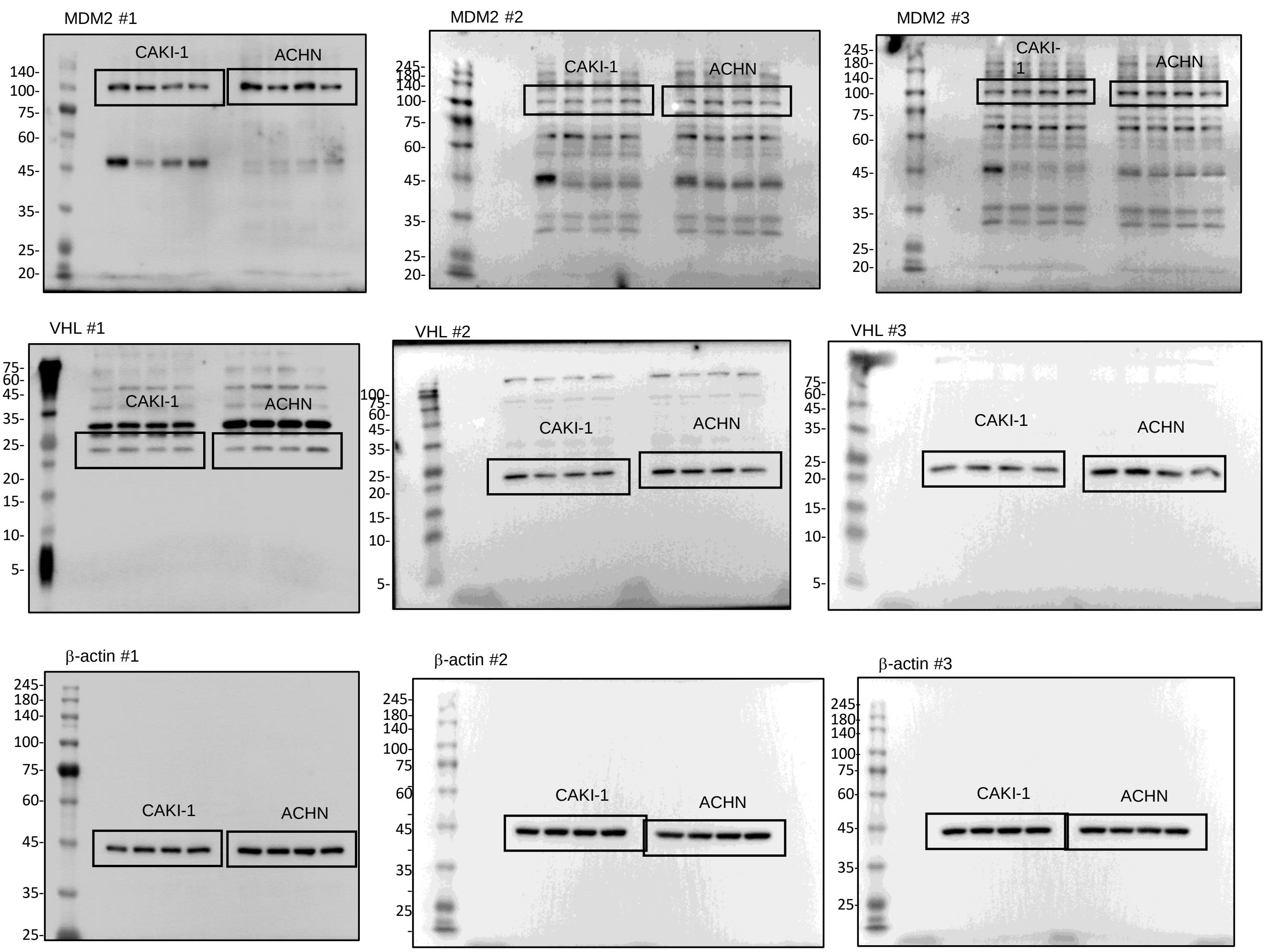

Supplementary Figure 2. Full gel images of western blotting

Supplementary Figure 2

Figure 2E

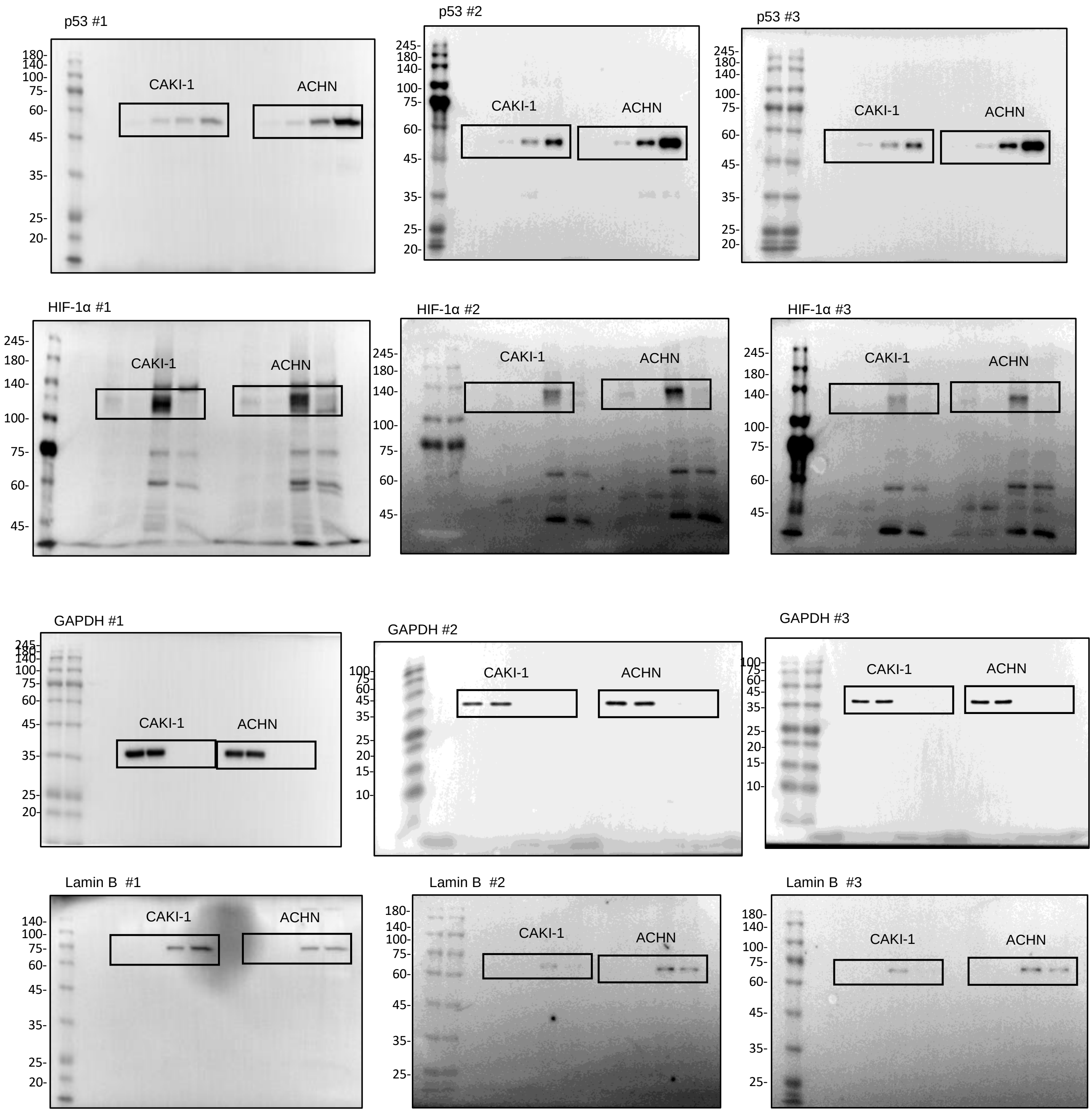

Supplementary Figure 2. Full gel images of western blotting

Supplementary Figure 2

Figure 3A

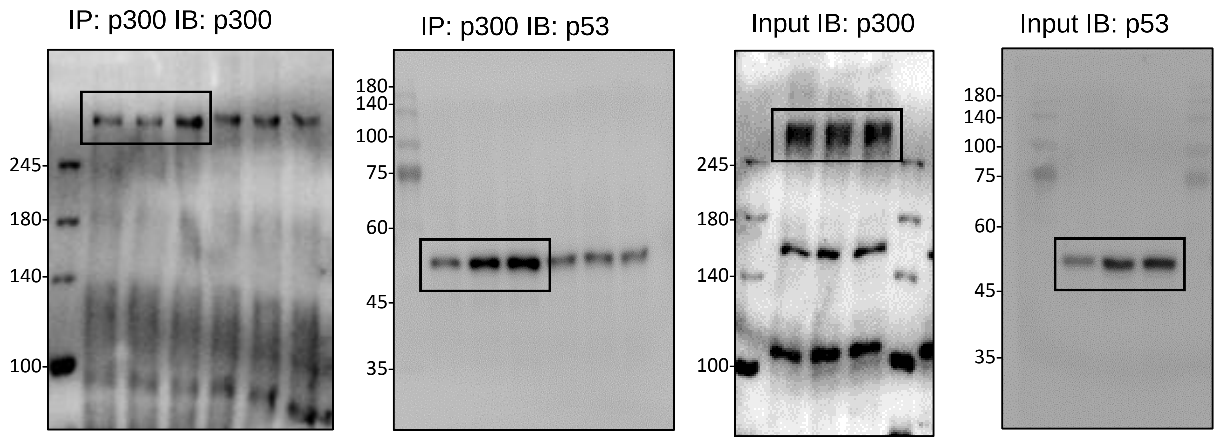

Supplementary Figure 2. Full gel images of western blotting

Supplementary Figure 3

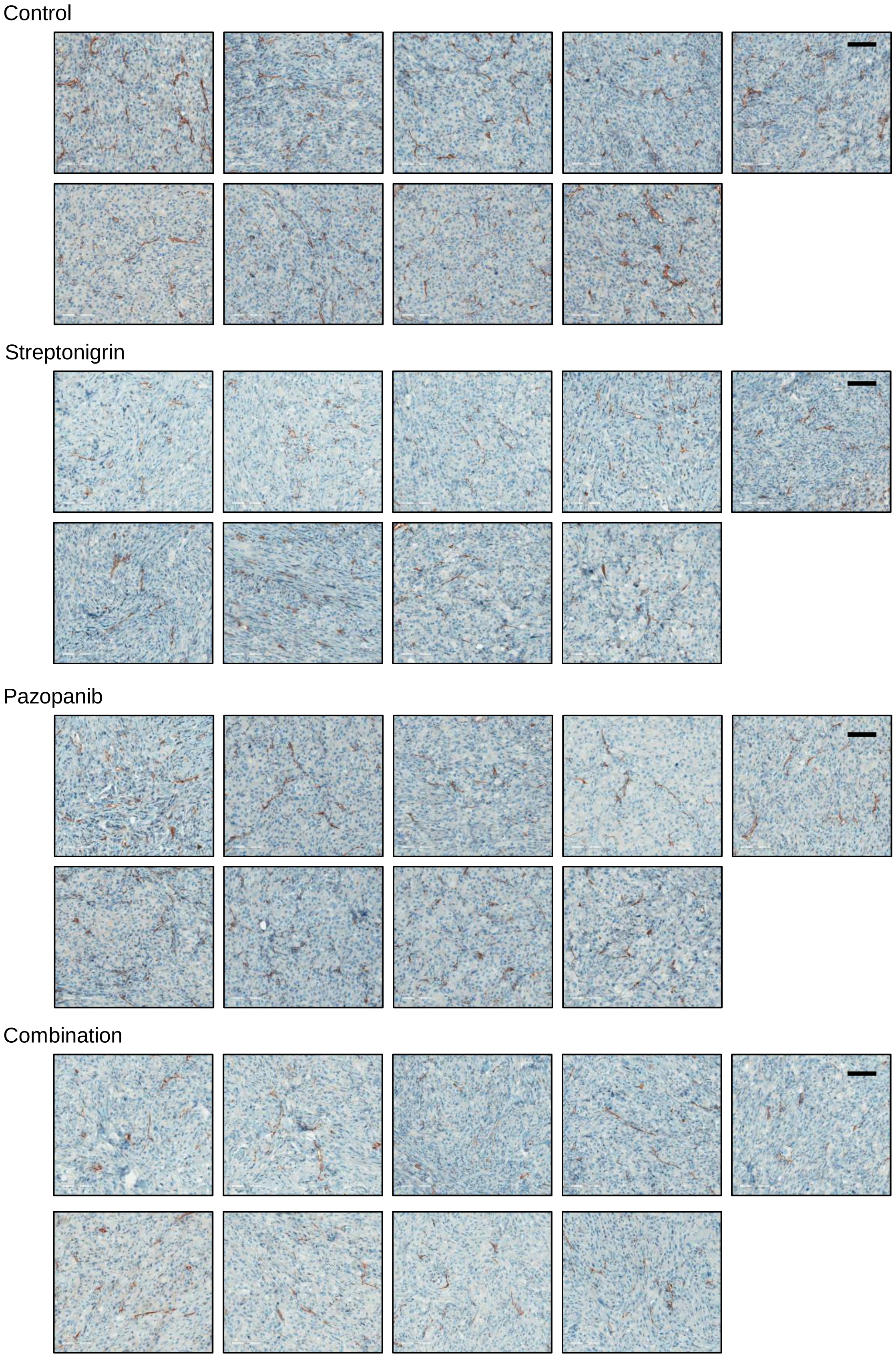

Supplementary Figure 3. Immunohistochemical staining of CD31 in CAKI-1 tumor xenograft. Scale bar = 100 μm.

Supplementary Table 1

Age	Sex	Organ	Diagnosis	TNM
59	Male	Kidney	renal cell carcinoma, clear cell type	T2aN0M0
67	Female	Kidney	renal cell carcinoma, clear cell and granular cell type	T2aNXM0
65	Male	Kidney	renal cell carcinoma, clear cell and granular cell type	T2bNXM0
59	Male	Kidney	renal cell carcinoma, clear cell and granular cell type	T1bNXM0
56	Male	Kidney	renal cell carcinoma, clear cell and pseudosarcomatous type	T1bN0M1
42	Female	Kidney	renal cell carcinoma, clear cell type	T3aNXM0
50	Male	Kidney	renal cell carcinoma, clear cell type	T1aN0M0
60	Female	Kidney	renal cell carcinoma, clear cell type	T3aN0M0
69	Female	Kidney	renal cell carcinoma, clear cell type	T2aNXM0
54	Male	Kidney	renal cell carcinoma, clear cell type	T1bNXM0
43	Female	Kidney	renal cell carcinoma, clear cell type	T1aN0M0
53	Male	Kidney	renal cell carcinoma, clear cell type	T3aN0M1
59	Male	Kidney	renal cell carcinoma, clear cell type	T3aN0M0
61	Male	Kidney	renal cell carcinoma, collecting duct type	T1aNXM0
40	Male	Kidney	renal cell carcinoma, clear cell type	T1aNXM0
64	Male	Kidney	renal cell carcinoma, clear cell type	T1bN0M0
37	Male	Kidney	renal cell carcinoma, collecting duct type	T2bN0M0
65	Male	Kidney	renal cell carcinoma, papillary type	T1bNXM0
74	Male	Kidney	renal cell carcinoma, clear cell type	T3aN0M0
67	Male	Kidney	renal cell carcinoma, clear cell type	T4NXM0
50	Male	Kidney	renal cell carcinoma	T2aN0M0
74	Male	Kidney	renal cell carcinoma, clear cell type	T3aN0M0
62	Male	Kidney	renal cell carcinoma, clear cell and granular cell type	T3aN0M0
45	Male	Kidney	renal cell carcinoma, clear cell type	T1bN0M0
62	Male	Kidney	renal cell carcinoma	T3aN1M0
53	Male	Kidney	renal cell carcinoma, clear cell type	T1bNXM0
64	Male	Kidney	renal cell carcinoma, clear cell type	T1bNXM0
72	Male	Kidney	renal cell carcinoma, clear cell type	T1aNXM0
62	Female	Kidney	renal cell carcinoma, clear cell type	T3aN0M0
56	Male	Kidney	renal cell carcinoma, clear cell type	T3aN0M0
58	Male	Kidney	renal cell carcinoma	T2aN0M0
57	Male	Kidney	renal cell carcinoma, clear cell and granular cell type	T3bN0M0
43	Female	Kidney	renal cell carcinoma, clear cell type	T1bN0M0
52	Female	Kidney	renal cell carcinoma, granular cell type	T1bN0M0
64	Male	Kidney	renal cell carcinoma, granular cell type	T3aN0M0
57	Male	Kidney	renal cell carcinoma, clear cell type	T1bN0M0
59	Male	Kidney	renal cell carcinoma, clear cell and granular cell type	T2aNXM0
47	Male	Kidney	renal cell carcinoma, clear cell type	T1aNXM0
77	Male	Kidney	renal cell carcinoma, clear cell type	T1aN0M0
47	Female	Kidney	renal cell carcinoma, clear cell and granular cell type	T2aNXM0
55	Female	Kidney	renal cell carcinoma, clear cell type	T3aN0M0

Supplementary table 1. Information of human kidney cancer tissue array. Tissues diagnosis were primary kidney renal cell carcinoma, clear cell type or granular cell type. Age was from 37 to 77. Gender was male or female. TNM stage were referred to the AJCC Cancer Staging Manual (7th Edition).
